# Blood glucose target in acute phase suggested by the analysis of the relationship between blood glucose profile and the severity of the diseases

**DOI:** 10.1186/cc13636

**Published:** 2014-03-17

**Authors:** M Hoshino, Y Haraguchi, K Oda, S Kajiwara

**Affiliations:** 1 Shisei Hospital, Saitama, Japan; 2 Keiyo Hospital, Tokyo, Japan

## Introduction

Blood glucose (BG) control in acute illness improves outcome. However, how to manage BG levels, or BG target, is not clearly elucidated. In this study, the BG target was suggested by the analysis of the relationship between BG profile and the severity of the diseases.

## Methods

Ninety-six patients were studied. The following parameters were calculated during the first week after ICU admission. (1) Maximum value of SOFA score (SOFAmax). (2) Mean, standard deviation, maximum, minimum, and difference of BG levels (BGm, BGsd, BGmax, BGmin, BGd (BGmax - BGmin), respectively). BG levels were measured basically every 6 hours. (3) Correlation between SOFAmax and BG parameters using two-dimensional (correlation coefficient r_t_) and linear regression analysis (r_l_).

## Results

(1) Mortality of the patients with SOFAmax 0 to 3, 4 to 5, 6 to 7, 8 to 9, and 10 or more were 0%, 14%, 23%, 40%, and 89%, respectively. (2) r_t _and r_l _(r_t_/r_l_) between SOFAmax and BG parameters: BGsd (0.49/0.36), BGmax (0.47/0.32), BGm (0.45/0.23), BGd (0.44/0.32), and BGmin (0.25/0.06). (3) BG ranges that indicate SOFAmax less than 5 calculated from the two-dimensional correlation curves between SOFAmax and BGsd, BGmax, BGm, and BGd were 35 ± 25, 250 ± 112, 150 ± 33, and 156 ± 115 mg/dl, respectively.

## Conclusion

BG parameters except BGmin were two-dimensionally related to the severity. Therefore, a part of the severe patients seemed to have lower BG levels and lower BG variability. Targeting those BG parameters in early phase within the abovementioned levels was considered to link to better outcome.

